# Acupuncture and electroacupuncture for cyclic vomiting syndrome with tachygastria in an adult: A case report

**DOI:** 10.1097/MD.0000000000040830

**Published:** 2024-12-20

**Authors:** Na-Yeon Ha, Jinsung Kim

**Affiliations:** a Division of Digestive Diseases, Department of Korean Internal Medicine, Kyung Hee University College of Korean Medicine, Kyung Hee University Korean Medicine Hospital, Seoul, Republic of Korea.

**Keywords:** acupuncture, case report, cyclic vomiting syndrome, electroacupuncture, nausea

## Abstract

**Rationale:**

Cyclic vomiting syndrome (CVS) is characterized by recurrent episodes of acute vomiting lasting <1 week, occurring independently and chronically. Management typically involves lifestyle interventions, supportive care, and preventative medication. In rare cases, CVS persists for decades in adults, requiring a multidisciplinary approach to improve symptoms and quality of life.

**Patient concerns:**

A 30-year-old male patient presented with a 7-year history of recurrent nausea and vomiting, which initially began in 2017. The episodes occurred without identifiable triggers such as alcohol or overeating, increasing in frequency from once every 4 months to 2 months. He expressed fear of severe deterioration, impacting his ability to eat freely, work, and enjoy his hobbies.

**Diagnoses:**

He was diagnosed with CVS based on the Rome criteria. Cutaneous electrogastrography showed postprandial power decrease and tachygastria.

**Interventions:**

Regular acupuncture treatments, including electroacupuncture at ST36 with 3 Hz, were administered twice a week for 3 weeks, followed by weekly sessions for 5 additional weeks.

**Outcomes:**

Over 11 treatment sessions across 8 weeks, no nausea or vomiting was observed. The visual analog scale (VAS) score for subjective gastrointestinal discomfort decreased from 67 to 0 after 4 weeks. The Nausea Severity Scale (NSS) score dropped from 14 to 0 after 4 weeks. The Functional Dyspepsia-related QoL score decreased from 16 to 0 after 8 weeks. The Nepean Dyspepsia Index-Korean version decreased from 80 to 8 after 8 weeks. Furthermore, the patient expressed high satisfaction with the treatment, and no adverse events were observed.

**Lessons:**

Acupuncture offers a significant and safe approach to relieving symptoms and enhancing the quality of life for patients with nausea and vomiting. Although this is a single case report, the findings suggest that acupuncture can improve treatment compliance and manage symptoms in adults with CVS. Further research, including clinical trials, is required to confirm these findings and understand the underlying mechanisms.

## 1. Introduction

Nausea is an unpleasant sensation often associated with a feeling of imminent vomiting, which may occur independently or precede the vigorous expulsion of stomach contents. According to the 2016 Rome IV criteria,^[1]^ chronic nausea and vomiting syndrome (CNVS), cyclic vomiting syndrome (CVS), and cannabinoid hyperemesis syndrome (CHS) are distinct functional gastroduodenal disorders without observable organic causes. CVS typically presents with stereotypical episodes characterized by an acute onset and a duration of <1 week. This condition manifests as at least 3 individual vomiting episodes occurring at intervals of at least 1 week over a year, with at least 2 cycles over 6 months. Although vomiting is absent between episodes, mild complaints may occur during symptom-free periods, and a personal or family history of migraines may support the diagnosis of CVS. CNVS is marked by persistent nausea and/or vomiting episodes without specific timing patterns, whereas CHS occurs after prolonged cannabis use, with symptoms improving once cannabis use stops. It is crucial to distinguish CVS from other nausea and vomiting disorders to ensure effective management.^[2]^

The prevalence of CVS in children is approximately 2%,^[3]^ with an incidence rate of around 3 cases per 100,000 individuals.^[4]^ However, its prevalence in adults is about 1.2%.^[5]^ Although CVS is less common in adults, it tends to involve longer and more frequent episodes. The clinical presentation of adult CVS primarily includes prodromal symptoms such as anorexia, epigastric pain, and sweating.^[6]^ Vomiting typically begins early in the morning and is accompanied by abdominal pain, with a high frequency of up to 8 episodes per hour. Symptoms often occur suddenly but may be triggered by factors like psychological stress, excitement, fatigue, infection, and the menstrual cycle.^[7]^

Given the complexity and uncertainty surrounding its pathophysiology, optimal diagnostic procedures and treatment modalities for CVS have not yet been established beyond lifestyle interventions and symptom-based medication prescriptions. To address these challenges, a multidisciplinary approach and non-pharmacological treatment methods are actively advocated in the management of CVS. These strategies aim to directly address symptoms, alleviate anxiety caused by uncertainty, and improve overall quality of life.^[8,9]^ Consequently, there is increasing attention on complementary and alternative medicines for treating CVS. Due to the difficulty in explaining the disease process of CVS through a single pathophysiology, alternative therapies for nausea and vomiting, such as acupuncture at PC6, have been explored.^[10]^ Acupuncture has previously been reported as a potential complementary treatment effective for nausea and vomiting.^[11]^ However, robust clinical data demonstrating its efficacy in clinical practice are lacking, highlighting the necessity for high-quality research to validate the treatment outcomes.

In this report, we describe the case of an adult diagnosed with CVS due to chronic periodic vomiting who underwent outpatient treatment with acupuncture. This article offers a detailed account of the patient’s symptoms, treatment protocols, and results.

## 2. Case presentation

A 30-year-old male patient presented with recurrent episodes of sudden vomiting since 2017. He reported episodes of vomiting once every 2 to 3 months, averaging 4 to 5 episodes annually. Initially, vomiting episodes lasted 5 days but were typically reduced to 3 days with prompt medical intervention at a local clinic. Each episode involved persistent vomiting for 3 to 5 days, occurring more than twenty times daily severely impairing his ability to eat and work. Chronic digestive discomfort and occasional heartburn before sleep were also reported, although there was no abdominal pain, headache, or other pain types. Episodes primarily occurred at night, and no specific triggers were identified. Prodromal symptoms included severe nausea rated 6 on a numeric scale, significantly affecting daily activities. The patient was a nonsmoker and abstained from alcohol, with no significant family history.

**Figure 1.**
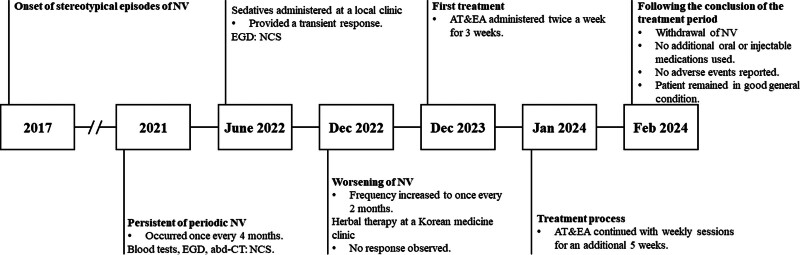
A brief timeline of clinical events. abd-CT = abdominal computed tomography, AT = acupuncture treatment, EA = electroacupuncture, EGD = esophagogastroduodenoscopy, NCS = not clinically significant, NV = nausea and vomiting.

Initially, the vomiting occurred annually, but recently the frequency increased to every 2 months. Despite undergoing blood tests, esophagogastroduodenoscopy, and abdominal computed tomography, no clinically significant findings were revealed. Partial symptom relief was achieved with sedatives at a local clinic. The patient received only herbal therapy at a Korean medicine clinic, but no improvement was noted, leading to the discontinuation of treatment. The recurring symptoms significantly affected his daily life, prompting him to seek further treatment. The timeline of clinical events is presented in Figure 1.

The patient reported a poor appetite and frequent indigestion. Bowel movements were normal, but occasional heartburn before bedtime disrupted his sleep. The unpredictability and fear of episodes caused anxiety and irritability, complicating his eating habits. A symptom-related questionnaire indicated a visual analog scale (VAS, 0–100 mm) score of 67 for nausea and vomiting, suggesting moderate-to-severe discomfort. The patient experienced persistent postprandial fullness and early satiety for over 3 days weekly in the past 6 months.

In the year preceding presentation, the patient experienced vomiting episodes 4 times, with 3 episodes occurring at intervals of at least 1 week in the last 6 months. These episodes followed a typical pattern of sudden onset, lasting <1 week. Although vomiting ceased between episodes, mild symptoms like indigestion persisted. CVNS was ruled out due to the presence of distinct temporal phases, which are not typical of CVNS. Additionally, CHS was excluded because the patient’s symptoms were unrelated to cannabis use. Despite extensive testing, no alternative diagnosis was established. Based on the Rome criteria, the patient was diagnosed with moderate-to-severe CVS (Table 1).^[1]^

**Table 1:** Rome IV criteria for the diagnosis of CVS.

Diagnostic criteria for CVS	Signs and symptoms observed in the patient
Must include all of the following:	
Stereotypical episodes of vomiting regarding onset (acute) and duration (<1 wk)	Yes
1. At least 3 discrete episodes in the past yr and 2 episodes in the past 6 mo, occurring at least 1 wk apart	4 discrete episodes in the past yr and 3 episodes in the past 6 mo
2. Absence of vomiting between episodes, but other milder symptoms can be observed between cycles	Indigestion and acid regurgitation
Supportive remarks:	
History or family history of migraine headaches	No

Abbreviation: CVS = cyclic vomiting syndrome.

**Figure 2.**
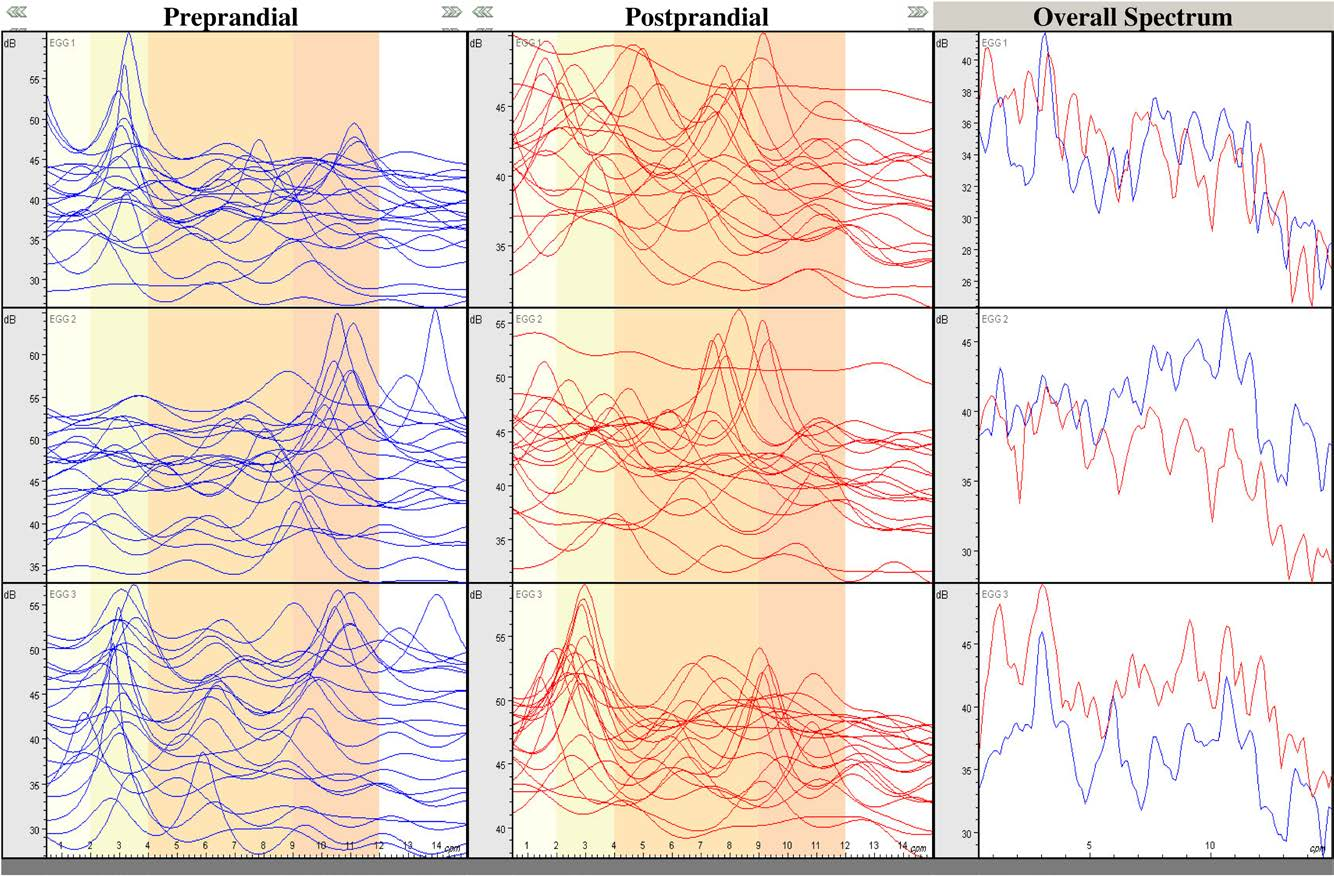
Spectral analysis of electrogastrography in the case.

Before initiating regular acupuncture treatment, electrogastrography (EGG) was performed to analyze the patient’s gastric myoelectrical activity (GMA).^[12]^ The procedure involved fasting for more than 8 hours, applying gel to the upper abdominal skin, and attaching 3 active electrodes. Multichannel EGG (Polygraf ID, Medtronic A/S, Skovlunde, Denmark) recorded measurements for 20 minutes before and after consuming standard nutritional drinks. Analysis focused on dominant frequency (cycles per minute, CPM) and power (dB), EGG classification percentages, and power ratio (Fig. 2). The dominant postprandial frequency was 3.2 CPM, within the normal range. However, EGG classification revealed normal gastria of 70% or more in only 1 channel (channel 3) at 75.0%, while the other 2 channels showed 60.0% and 55.0%, respectively. Tachygastria was notably high at 25.0% in channels 1 and 2. Previous studies indicate that tachygastria exceeding 25% suggests total inhibition of antral contraction,^[13]^ commonly observed in gastric motility disorders and patients with unexplained nausea and vomiting.^[14,15]^ Additionally, the power ratio decreased to <1 in channels 1 and 2, indicating reduced gastric contractility (Table 2).^[12]^

**Table 2:** Results of the electrogastrography analysis.

Overall analysis	Preprandial			Postprandial		
Dominant frequency (CPM)	3.0			3.2		
Dominant power (dB)	42.6			41.8		

Percentage electrogastrogram classification	Ch1	Ch2	Ch3	Ch1	Ch2	Ch3
% Normal	71.4	52.4	81.0	60.0	55.0	75.0
% Bradygastria	0.0	0.0	4.8	0.0	0.0	5.0
% Tachygastria	14.3	19.0	14.3	25.0	25.0	0.0
% Arrhythmic	14.3	28.6	0.0	15.0	20.0	20.0

Power ratio calculations	Ch1		Ch2		Ch3	
Postprandial-Preprandial	0.8		0.8		2.4	

Abbreviations: Ch = channel, CPM = cycle per minute.

## 3. Clinical course

**Figure 3.**
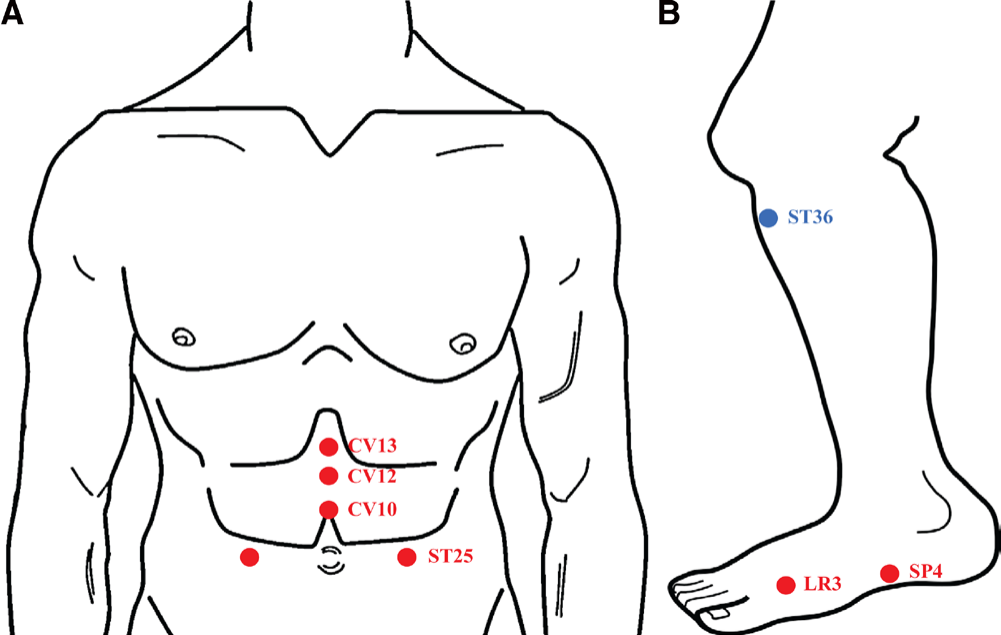
Acupoints used for treatment. (A) Abdomen; (B) lower leg. Red and blue dots represent target sites for acupuncture and electroacupuncture, respectively.

Acupuncture treatment, including electroacupuncture, was initiated with the aim of reducing abdominal discomfort likely stemming from gastric dysmotility. Disposable stainless-steel needles (0.25 mm × 40 mm, Dong Bang Medical Co., Ltd., Boryung, Korea) were used for the procedure. The needles were inserted at specific acupoints on both lower limbs (ST36, LR3, and SP4) and the abdomen (CV13, CV12, CV10, and ST25) to a depth of 1 to 2 cm and retained for 20 minutes (Fig. 3). Electroacupuncture was also administered, pairing needles at ST36 with a frequency of 3 Hz using an ES160 digital stimulator (ITO Co., Ltd., Saitama, Japan). The current intensity was adjusted to 2 to 6 mA. The acupoint stimulation was conducted twice a week for 3 weeks, followed by weekly sessions for an additional 5 weeks.

## 4. Treatment response

The VAS, Nausea Severity Scale (NSS), Nepean Dyspepsia Index-Korean version (NDI-K), and Functional Dyspepsia-related Quality of Life (FD-QoL) were used to evaluate the frequency and intensity of nausea and vomiting symptoms and their overall impact on quality of life (Table 3).

**Table 3:** Overall treatment procedures in the present case.

Procedures	Treatment period										
	Visit 1	Visit 2	Visit 3	Visit 4	Visit 5	Visit 6	Visit 7	Visit 8	Visit 9	Visit 10	Visit 11
Date	December 5, 2023	December 6, 2023	December 12, 2023	December 15, 2023	December 19, 2023	December 21, 2023	December 26, 2023	January 2, 2024	January 8, 2024	January 16, 2024	February 2, 2024
Timepoints	Week 0		Week 1		Week 2		Week 3	Week 4	Week 5	Week 6	Week 8
*History taking*											
Demographic information	X										
Medical history	X										
*Measurements*											
Height/Weight/BMI	X										
Vital signs	X										
Electrogastrography		X									
*Interventions*											
Acupuncture & EA	X	X	X	X	X	X	X	X	X	X	X
*Assessments*											
VAS	X		X					X			X
NSS	X							X			X
NDI-K	X							X			X
FD-QoL	X							X			X
Adverse events	X	X	X	X	X	X	X	X	X	X	X
Patient’s comments:	Severe N/V/I.	N/B/R before bedtime	NSTR	No N/V/B/I; Alleviation of anxiety about symptom recurrence.	Only I after overeating 3 d ago; No N/V/B.	No discomfort.	Only postprandial fullness after overeating in the evening; No N/V/B.	No discomfort	No discomfort	No discomfort	Much improved; Only feel mild fullness and heaviness after overeating; No N/V/B.

Abbreviations: B = heartburn, BMI = body mass index, EA = electroacupuncture, FD-QoL = functional dyspepsia-related quality of life, I = indigestion, N = nausea, NSS = nausea severity scale, NSTR = nothing significant to report, NDI-K = Nepean dyspepsia index-Korean version, R = acid reflux, V = vomiting, VAS = visual analog scale.

To objectively assess the patient’s subjective discomfort and the severity of nausea and vomiting symptoms, a VAS was used, where the patient marked his discomfort on a 100 mm scale. The NSS evaluates the frequency, daily incidence, duration, and severity of nausea over the preceding 2 weeks, with a total score calculated as the sum of 4 items (0–4 points).^[16]^ The NDI-K measures alterations in upper GI symptoms over the past 2 weeks. This scale assesses the frequency, severity, and degree of discomfort associated with 15 symptoms (pain, discomfort, burning sensation, cramps, pressure, and bloating in the upper abdomen, heartburn, chest pain, regurgitation, inability to finish meals, postprandial fullness, nausea, vomiting, belching, and bad breath) on a scale of 0 to 5, with the final score derived from the sum of all individual scores.^[17]^ The FD-QoL scale is a 5-point tool comprising 21 items developed to evaluate the psychological, role-functioning, eating, and liveliness statuses of patients with FD. Scores for each domain and the total score were calculated, with lower scores indicating a higher quality of life.^[18]^

**Figure 4.**
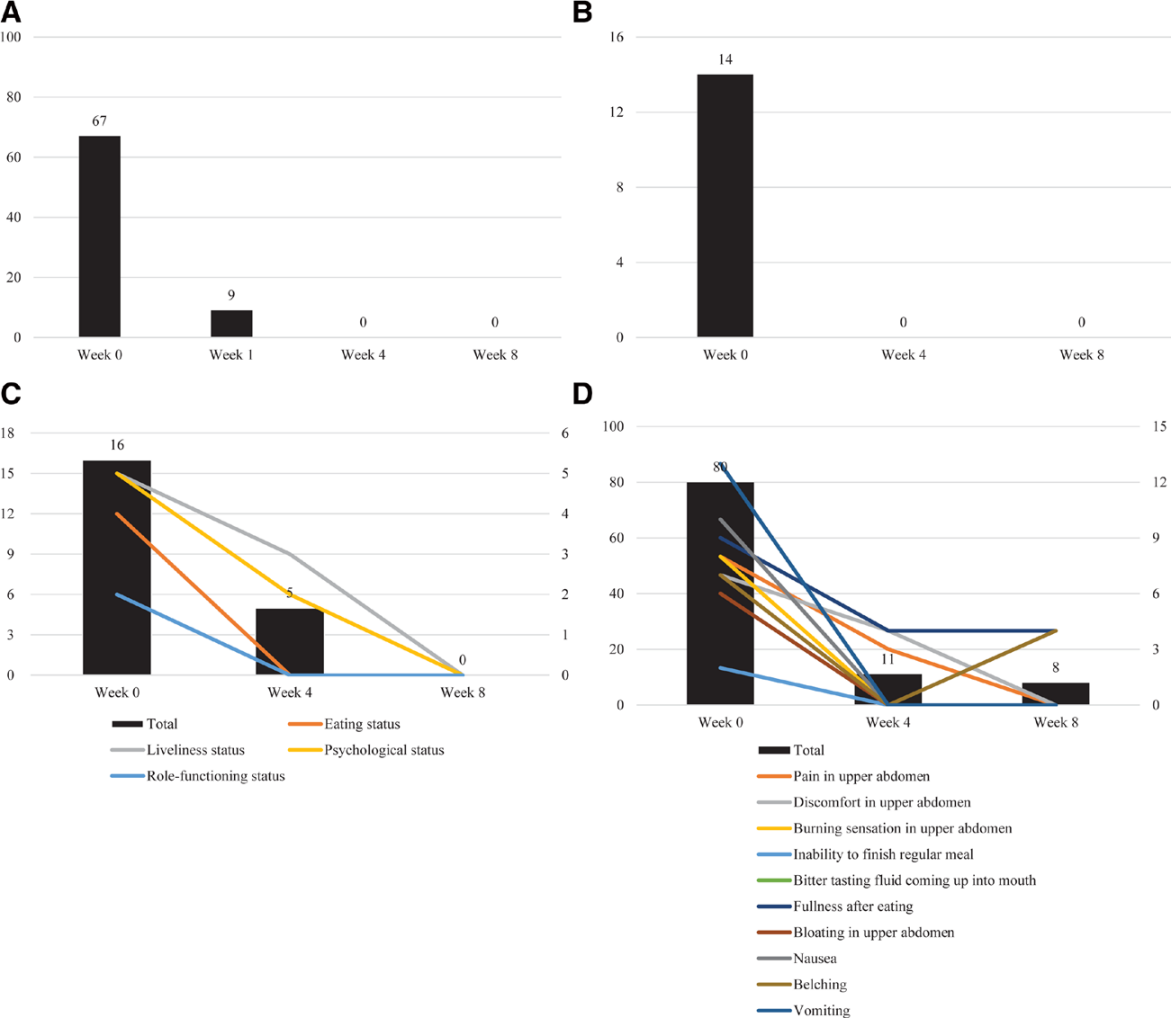
Changes in assessment scale scores. (A) Visual analogue scale; (B) nausea severity scale; (C) functional dyspepsia-related quality of life; (D) nepean dyspepsia index-Korean version.

Throughout the 8-week treatment course, the VAS was evaluated 4 times (weeks 0, 1, 4, and 8), while the NSS, NDI-K, and FD-QoL were assessed 3 times (weeks 0, 4, and 8). The VAS score decreased from 67 to 9 after 1 week of treatment and reached 0 after 4 weeks. The NSS score decreased from 14 at baseline to 0 after 4 weeks of treatment. The FD-QoL score decreased from 16 to 5 after 4 weeks and to 0 after 8 weeks. The NDI-K scores decreased from a baseline of 80 to 11 after 4 weeks and to 8 after 8 weeks. (Fig. 4). No nausea or vomiting was observed during the treatment period.

Subjective comments on GI discomfort were recorded throughout the treatment. At the initial visit, the patient reported severe nausea and vomiting symptoms occurring 6 days prior, leading to a notable decrease in quality of life. He also expressed fear of worsening symptoms and difficulty eating normally. However, after 1 month of treatment, the patient no longer experienced symptoms such as nausea, vomiting, or heartburn, except for occasional postprandial fullness. After 2 months of treatment, there was substantial improvement in overall symptoms, with the patient reporting a greatly improved quality of life and only mild feelings of fullness and discomfort when eating excessively. No additional symptomatic medications or injection therapies were used posttreatment, and no adverse events were observed during the treatment period.

## 5. Discussion

CVS is a chronic condition characterized by prolonged and frequent episodes of vomiting that significantly affect the overall quality of life. This disorder can persist for several months or even decades. Diagnosing CVS is often time-consuming due to its rarity in adults.^[6,7]^ The etiology of CVS remains unclear, with gastric emptying considered an important factor, prompting the use of cutaneous EGG and gastric emptying scintigraphy for gastric motility studies.^[14]^ Compared to other functional GI disorders, abnormalities in EGG, such as tachygastria and decreased postprandial power, are common among patients with CVS.^[19]^

The pathophysiology of CVS is multifactorial, involving several pathways. CVS is associated with psychosocial factors, neurohumoral contributors, cannabis use, lifestyle habits,^[20]^ autonomic nervous system dysfunction,^[21]^ excessive activation of the hypothalamic-pituitary-adrenal axis, and impaired mitochondrial energy production.^[22]^ Additionally, evidence suggests that CVS is a neurogenic disorder sharing neurobiological mechanisms with chronic migraine, epilepsy, and panic disorders, leading to the proposal of the CVS threshold as a key concept for understanding vomiting attacks in adults with CVS.^[23]^

Several strategies for effectively managing CVS have been recommended, including: lifestyle interventions to reduce the risk of triggering episodes and improve self-efficacy and quality of life, such as adequate hydration, regular meals, exercise, good sleep hygiene, and stress management; supportive care during severe vomiting episodes, which may involve emergency room or hospital admission, intravenous hydration, antiemetic medications, and sedating agents such as triptans, ondansetron, phenothiazines, antihistamines, and benzodiazepines; and prophylactic medication to prevent or mitigate episodes and prevent subsequent episodes, including tricyclic antidepressants, topiramate, and coenzyme Q10.^[6,24]^ Most patients with CVS experience a gradual decrease in symptoms over time. However, some adult patients may experience worsening symptoms without symptom-free intervals, with daily nausea and vomiting becoming more severe.^[2]^

Before acupuncture treatment, the patient underwent conventional drug and injection therapies at various hospitals. However, periodic vomiting, heartburn, and indigestion persisted. The patient exhibited a typical 4-phase CVS pattern.^[25]^ During the symptom-free phase, the patient was able to lead a normal daily life with mild indigestion. The prodromal phase was characterized by varying degrees of nausea and malaise, often severe enough to impair daily activities. The emetic phase involved persistent severe nausea and repetitive vomiting, sometimes occurring up to 20 to 30 times a day, lasting 3 to 5 days, and leading to severe dehydration. The recovery phase presented a decrease in vomiting episodes, improvement in symptoms, and a return to oral intake and vitality. These phases necessitate appropriate treatment, and the uncertainty of vomiting episodes and fear of relapse significantly impair the patient’s quality of life, causing notable anxiety and irritability. Specifically, during the emetic phase, when severe vomiting makes eating and daily activities impossible, close monitoring, ongoing treatment, daily management, and long-term follow-up are essential.

The patient with CVS in this case exhibited significant improvements in the frequency and intensity of nausea and vomiting symptoms, as well as in indigestion-related symptoms and quality of life scores, through acupuncture treatment. Additionally, the patient showed high treatment compliance without any adverse events and did not require sleep aids or intravenous therapy, which had previously been administered as needed.

The efficacy of acupuncture and electroacupuncture in treating nausea and vomiting has been well-documented in numerous randomized controlled trials (RCTs).^[26–30]^ These studies demonstrate its effectiveness across a variety of conditions such as postoperative, chemotherapy-induced, and pregnancy-related. The underlying mechanisms by which acupuncture influences gastrointestinal motility are explained through neural, humoral, and serotonin pathways.^[31]^ Electroacupuncture has consistently been reported to increase the proportion of regular slow waves and influence their frequency.^[32]^ Electroacupuncture stimulation at the ST36 acupoint increases the regularity of gastric slow waves and accelerates gastric emptying through opioid and vagal pathways.^[33,34]^ Additionally, it is associated with increased GMA mediated by cholinergic nerves, which are related to gastric contraction.^[35]^ These mechanisms may explain the therapeutic effects of acupuncture in patients with nausea and vomiting. The acupoints used in this case, including ST36, CV12, LR3, ST25, and SP4, are commonly used in essential acupoint combinations for patients with FD,^[36]^ accounting for the effective response observed in this patient, who showed findings of gastric dysrhythmia on EGG examination. Another advantage of acupuncture is its relatively low and largely nonserious side effects, with its safety proven in several studies.^[11]^

This case study has some limitations. Long-term observation and evaluation of the patient’s condition were not feasible, and an assessment with EGG after treatment could not be conducted. This case report highlights the need for large-scale, high-quality RCTs to explore the efficacy and underlying mechanisms of acupuncture in treating CVS. Nevertheless, it represents the first documentation of the use of acupuncture for adult CVS, particularly in a case accompanied by tachygastria on EGG. The report provides evidence supporting acupuncture as a viable clinical option for managing CVS in adults, demonstrating improvement in GI symptoms during asymptomatic periods, with high compliance, no adverse reactions, and an extension in the interval between vomiting episodes.

## 6. Conclusions

Although this study is limited to a single case report, the functional improvement observed after 8 weeks of acupuncture and electroacupuncture demonstrated significant benefits. Standardized measures indicated reductions in nausea and vomiting, effective management of GI symptoms, and improved QoL in an adult patient with CVS, all without severe adverse effects. While definitive conclusions cannot be drawn from a single uncontrolled case report, these observations suggest that acupuncture is a safe and effective approach for managing adult CVS.

## Author contributions

**Conceptualization:** Na-Yeon Ha.

**Investigation:** Na-Yeon Ha.

**Supervision:** Jinsung Kim.

**Visualization:** Na-Yeon Ha.

**Writing – original draft:** Na-Yeon Ha.

**Writing – review & editing:** Jinsung Kim.
